# Supplementary material for: Empirical methods for controlling false positives and estimating confidence in ChIP-Seq peaks
Source: BMC Bioinformatics. 2008 Dec 5;9:523. doi: 10.1186/1471-2105-9-523 (PMC2628906; doi:10.1186/1471-2105-9-523)
Supplement: Additional File 1 — A variety of html documents from the USeq web site detailing the available applications, their best usage, output file type descriptions, command line menus, etc. [file 1471-2105-9-523-S1.zip › USeqUserGuides/JavaDoc.zip!/JavaDoc/util/amazon/S3CodeSamples.html]

S3CodeSamples


|  |  |  |
| --- | --- | --- |
| **Overview** \| **Package** \| **Class** \| **Use** \| **Tree** \| **Deprecated** \| **Index** \| **Help** | |  |
| PREV CLASS   **NEXT CLASS** | **FRAMES**    **NO FRAMES**     **All Classes** |  |
| SUMMARY: NESTED \| FIELD \| CONSTR \| METHOD | DETAIL: FIELD \| CONSTR \| METHOD |  |


---


## util.amazon Class S3CodeSamples

```
java.lang.Object
  util.amazon.S3CodeSamples
```

---

``` public class S3CodeSamples extends java.lang.Object ```

---

| **Field Summary** | |
| --- | --- |
| `static java.lang.String` | `AWS_ACCESS_KEY_PROPERTY_NAME` |
| `static java.lang.String` | `AWS_SECRET_KEY_PROPERTY_NAME` |
| `static java.lang.String` | `SAMPLES_PROPERTIES_NAME` |


**Constructor Summary**

`S3CodeSamples()`


| **Method Summary** | |
| --- | --- |
| `static org.jets3t.service.security.AWSCredentials` | `loadAWSCredentials()`             Loads AWS Credentials from the file samples.properties (`SAMPLES_PROPERTIES_NAME`) that must be available in the classpath, and must contain settings awsAccessKey and awsSecretKey. |
| `static void` | `main(java.lang.String[] args)` |

**Methods inherited from class java.lang.Object**

`equals, getClass, hashCode, notify, notifyAll, toString, wait, wait, wait`

**Field Detail**

### SAMPLES\_PROPERTIES\_NAME

```
public static final java.lang.String SAMPLES_PROPERTIES_NAME
```

**See Also:**: Constant Field Values

---


### AWS\_ACCESS\_KEY\_PROPERTY\_NAME

```
public static final java.lang.String AWS_ACCESS_KEY_PROPERTY_NAME
```

**See Also:**: Constant Field Values

---


### AWS\_SECRET\_KEY\_PROPERTY\_NAME

```
public static final java.lang.String AWS_SECRET_KEY_PROPERTY_NAME
```

**See Also:**: Constant Field Values


**Constructor Detail**

### S3CodeSamples

```
public S3CodeSamples()
```


**Method Detail**

### main

```
public static void main(java.lang.String[] args)
                 throws java.lang.Exception
```

:   **Throws:**: `java.lang.Exception`

---


### loadAWSCredentials

```
public static org.jets3t.service.security.AWSCredentials loadAWSCredentials()
                                                                     throws java.io.IOException
```

:   Loads AWS Credentials from the file samples.properties
    (`SAMPLES_PROPERTIES_NAME`) that must be available in the
    classpath, and must contain settings awsAccessKey and
    awsSecretKey.

    :   **Returns:**: the AWS credentials loaded from the samples properties file. **Throws:**: `java.io.IOException`


---


|  |  |  |
| --- | --- | --- |
| **Overview** \| **Package** \| **Class** \| **Use** \| **Tree** \| **Deprecated** \| **Index** \| **Help** | |  |
| PREV CLASS   **NEXT CLASS** | **FRAMES**    **NO FRAMES**     **All Classes** |  |
| SUMMARY: NESTED \| FIELD \| CONSTR \| METHOD | DETAIL: FIELD \| CONSTR \| METHOD |  |


---
